# Adverse Childhood Experiences and Changing Levels of Psychosocial Distress Scores across Pregnancy in Kenyan Women

**DOI:** 10.3390/ijerph17103401

**Published:** 2020-05-13

**Authors:** Pauline Samia, Shahirose Premji, Farideh Tavangar, Ilona S. Yim, Sikolia Wanyonyi, Mohamoud Merali, Wangira Musana, Geoffrey Omuse, Ntonghanwah Forcheh, Aliyah Dosani, Nicole Letourneau, MIGHT Consortium

**Affiliations:** 1 Department of Pediatrics and Child Health, Aga Khan University Hospital, Nairobi 0100, Kenya; 2 School of Nursing, Faculty of Health, York University, Toronto, ON M3J 1P3, Canada; premjis@yorku.ca (S.P.); f.tavangar@gmail.com (F.T.); nforcheh@gmail.com (N.F.); MiGHT.CollaboratorsInResearch@gmail.com (MiGHT); 3 Department of Psychological Science, University of California Irvine, Irvine, CA 92697, USA; ilona.yim@uci.edu; 4 Department of Obstetrics and Gynaecology, Aga Khan University Hospital, Nairobi 00100, Kenya; sikolia.wanyonyi@aku.edu (S.W.); Wangira.Musana@aku.edu (W.M.); 5 Department of Counselling & Clinical Psychology, Aga Khan University Hospital, Nairobi 00100, Kenya; mohamoud.merali@aku.edu; 6 Department of Pathology, Aga Khan University Hospital, Nairobi 00100, Kenya; geoffrey.omuse@aku.edu; 7 School of Nursing and Midwifery, Mount Royal University, Calgary, AB T3E 6K6, Canada; adosani@mtroyal.ca; 8 Cumming School of Medicine (Community Health Sciences), University of Calgary, Calgary, AB T2N 1N4, Canada; 9 Faculty of Nursing and Cumming School of Medicine (Pediatrics, Psychiatry & Community Health Sciences), University of Calgary, Calgary, AB T2N 1N4, Canada; nicole.letourneau@ucalgary.ca

**Keywords:** adverse childhood experiences, depression, pregnancy-related anxiety, perceived stress, psychosocial/perinatal distress

## Abstract

Background: Adverse childhood experiences (ACEs) have been associated with deleterious effects on mental health in pregnancy. Methods: The ACE International Questionnaire (ACE-IQ) was used to measure neglect, abuse, and household dysfunction. Longitudinal mixed effect modelling was used to test the effect of ACEs on pregnancy-related anxiety, depressive symptoms, and perceived stress at two time points (12–19 and 22–29 weeks) during pregnancy. Results: A total of 215 women who were predominantly married (81%) and had attained tertiary education (96%) were enrolled. Total ACEs were significantly associated with depressive symptoms (r = 0.23, *p* < 0.05) and perceived stress (r = 0.18, *p* < 0.05). As depressive symptoms decreased, t (167) = −8.44, *p* < 0.001, perceived stress increased, t (167) = 4.60, *p* < 0.001, and pregnancy-related anxiety remained unchanged as pregnancy progressed. Contact sexual abuse (*p* < 0.01) and parental death or divorce (*p* = 0.01) were significantly associated with depression over time (*p* < 0.01). Total ACEs in this study were associated with depressive symptoms early but not late in pregnancy. Conclusions: Higher total ACEs were positively associated with depressive symptoms and perceived stress during pregnancy, suggesting that mental disorders may have an impact on pregnancy outcomes and ought to be addressed. Further validation of the Edinburgh Postnatal Depression Scale (EPDS) tool in local settings is required.

## 1. Background

Adverse childhood experiences (ACEs) are defined as traumatic experiences that occur before 18 years and include abuse, neglect, and household dysfunction [1,2]. ACEs constitute a massive stressor with long-lasting deleterious effects on mental and physical health [3,4], along with a strong graded relationship between ACEs and poor health outcomes [5,6,7]. ACEs have been shown to have negative effects on maternal reproductive health and pregnancy experiences such as reduced birth weight and gestational age (GA) [2,8,9,10,11]. Psychosocial distress, which describes symptoms of depression, pregnancy-related anxiety (PRA), and stress, has been reported to be a strong risk factor for poor pregnancy outcomes and postpartum depression [12,13,14]. Because prenatal depression, anxiety, and stress are also linked to poor health outcomes in offspring [15], understanding how ACEs impact mothers’ mental health in pregnancy is an important goal with implications for understanding the risk to both mother and child.

Most of the data linking ACEs and health outcomes originate from high-income contexts. There is evidence suggesting that mothers from Low and Middle Income countries (LMIC) suffer heightened vulnerability for ACEs, where chronic and co-occurring adversity may be more severe and longstanding [4,16,17]. Documented rates of childhood adversity in Kenya are high, with university students reporting the prevalence of physical abuse at 59% and neglect at 42% [18]. A nationally representative household survey reports 32% of females and 18% of males having experienced sexual violence, while 66% of females and 73% of males experienced physical violence [19]. In other LMIC, the prevalence of antenatal depression ranges from 18% to 25% [20,21,22], while that in high-income countries is much lower and spans 6% to 16% [23,24]. A study in South Africa found that maternal childhood trauma predicted greater maternal depressive symptoms and more negative child outcomes within the first six months post-partum [25]. Although pregnancy-related anxiety has not been extensively studied in LMIC, there is accumulating evidence suggesting that negative pregnancy outcomes associated with pregnancy-related anxiety are common in these settings [22,26,27,28].

Pregnant Kenyan adolescents who had experienced an adverse event or an extremely stressful life context were identified to be at higher risk for depression [29]. Another Kenyan study [4] showed that for women in rural regions, maternal ACEs are associated with negative maternal mental health outcomes, conditional on mothers’ levels of education, which were in turn associated with more child mental health problems. Similarly, in a comparative analysis of data obtained from women enrolled in two centers in Northern California’s non-profit integrated health delivery system, the association between ACEs and mental health was evident only among women with low resilience but not among women with high resilience [30].

In summary, studies evaluating the relationship between ACEs and psychosocial distress among pregnant women in LMIC are significantly limited despite a higher prevalence of depression and anxiety in these settings [31]. Furthermore, as optimal pregnancy mental health is linked to better health outcomes in offspring, we sought to examine the relationship between ACEs and pregnancy-related anxiety, depressive symptoms, and perceived stress (PS) during pregnancy among a sample of women from a low and middle income country to provide an opportunity to identify ACE-related risk factors to better inform future interventions and improve health outcomes.

## 2. Materials and Methods

### 2.1. Data and Sample

In this prospective study, pregnant women were recruited at antenatal clinic care visits between 12 and 19 weeks’ GA and were followed at two additional time points: between 22 and 29 weeks’ Gestational age (GA) and at delivery. The study took place over 12 months at the Aga Khan University Hospital, Nairobi (AKUH-N), a private not-for-profit institution primarily serving an urban population in Nairobi with an annual average of 2900 deliveries per year. Pregnant women aged 18 years and older, with a singleton pregnancy between 12 and 19 weeks’ GA at the time of recruitment, willing to return for an additional assessment 10 weeks later, who planned to deliver at AKUH-N, and who were able to speak English, were eligible to participate. Pregnancies were dated using the last menstrual period. Participants completed self-report questionnaires at the clinic, and text message reminders were sent to encourage women to return for follow-up visits 10 weeks later. Additional data were obtained from delivery and postpartum unit records at AKUH-N. All the study participants were above 18 years of age and provided written informed consent to participate in the study.

### 2.2. Measures

ACEs, a predictor variable, and the covariates discussed below were measured at the time of recruitment (i.e., 12–19 weeks’ GA). The three outcome variables (i.e., depressive symptoms, pregnancy-related anxiety, and perceived stress) were measured at the time of recruitment (12–19 weeks) and again 10 weeks later (22–29 weeks).

### 2.3. Predictor: ACEs

The Adverse Childhood Experiences International Questionnaire (ACE-IQ) is a 43-item questionnaire intended for use in adults. It assesses responses that are mapped onto the 13 most commonly examined ACEs including child maltreatment, violence, and broader experiences of household dysfunction (e.g., violence between parents, parental separation, and a household affected by substance misuse, mental illness, or criminal behavior) [32]. Each of the 13 ACE-IQ items was coded as 0 (did not occur during childhood) and 1 (occurred) and the resulting 13 binary responses for each woman were summed to obtain her aggregate score (range 0–13). Murphy et al. (2014) demonstrated excellent convergent validity and internal consistency (α = 0.88) of the ACE Questionnaire [33].

### 2.4. Outcome Variables

We assessed three outcome variables in this study: prenatal depressive symptoms, pregnancy-related anxiety, and perceived stress, which we collectively refer to as psychosocial distress.

The Edinburgh Postnatal Depression Scale (EPDS) was used to screen pregnant women for depression [34,35,36]. Respondents rated the presence and severity of each of ten depressive symptoms over the previous week. Item responses ranged from 0 to 3, resulting in a maximum score of 30. Scores above 13 are considered indicative of a depressive illness [34,35]. The reliability values of the EPDS, indicated by Cronbach’s α coefficient per trimester, have previously been validated as 0.82, 0.83, and 0.84, respectively [34].

For pregnancy-related anxiety, the PRA scale, a 10-item tool (4-point Likert scale) was used, which evaluates feelings related to health during pregnancy, the health of the fetus/infant, and labor and delivery, with scoring reversed where appropriate [27]. The sum of responses ranged from 0 to 30. Unlike the depression scale, the definition of high anxiety was not based on a cut-off value for the total score. Instead, women with three or more “very true” responses were considered as having high pregnancy-related anxiety. This scale has high internal consistency reliability (Cronbach’s α > 0.90) and has been utilized in similar studies [37].

Perceived stress was measured using the PS scale, a 10-item questionnaire using a 5-point Likert scale which measures the degree to which specific events occurring in the past month are viewed as stressful [38]. Each item was scored from 0 (no perceived stress) to 4 (high perceived stress) and the 10 items were summed to calculate the total score for each respondent (range from 0 to 40). We used a cut-off of 17 as indicative of high perceived stress [38]. Hence, respondents who scored 18 or more were classified as experiencing a high level of stress. The psychometric properties of the PSS-10 were originally evaluated in a large national sample by Cohen and Williamson who reported that scores on the PSS-10 demonstrated adequate internal consistency reliability (α = 0.78), and since then it has been found to have similar reliability in other studies [39].

### 2.5. Covariates

Potential covariates thought to moderate the relationship between ACEs and psychosocial distress during pregnancy were selected based on the strength of evidence from the literature, clinical judgement, and cultural considerations. Covariates were measured at baseline. A sociodemographic questionnaire elicited information from participants on age, marital status, ethnicity, level of education, occupation, annual household income, and information on selected risk behaviors such as alcohol intake and cigarette smoking.

### 2.6. Statistical Methods

In order to test whether the three psychosocial distress responses—pregnancy-related anxiety, EPDS, and PS—varied during pregnancy, we fitted mixed-effects models using the Restricted Maximum Likelihood Estimation Method (REML). In these models, psychosocial distress measures were used as dependent variables while the independent variables were time (0 for 12–19 weeks and 1 for 22–29 weeks) as a random factor, and ACE-IQ as a fixed factor.

Four models were considered for each dependent variable. Model 1 was fitted to investigate the effect of the overall ACE-IQ score on each individual psychosocial distress variable over time. A significant interaction effect between ACE-IQ and time was interpreted as evidence that the effect of ACE-IQ on the psychosocial distress changed during pregnancy. In model 2, the covariates were added to model 1 to investigate if any of them moderate the effects of ACE-IQ found in model 1. We utilized the Likelihood Ratio Test (LRT) and t-test to determine the overall significance of models and each model parameter respectively. Models 3 and 4 were similar to models 1 and 2 respectively in which the ACE-IQ score was replaced with the 13 ACE-IQ binary indicators and likelihood ratio criterion used to retain only significant variables in the final model. The analyses were performed in the R software (The R Foundation for Statistical Computing, Vienna, Austria).

## 3. Results

### 3.1. Preliminary Analyses

Participants were aged between 22 and 47 years (mean age was 30.6), predominantly married (80.9%), highly educated (95.8% attended college/university), and affluent (80.5% earned > Kenya Shillings 100,000). Almost half (48.8%) were non-government employees and a few of the women (4.7%) were students. Very few women reported drinking alcohol (2.8%), or cigarette smoking (0.5%). Detailed sociodemographic characteristics of the 215 women are shown in Table 1.

The most commonly reported ACE was emotional neglect (84.2%), followed by community violence (79.5%), and physical abuse (77.7%). Physical neglect (5.6%), incarcerated household member (3.3%), and mentally ill household member (2.3%) were endorsed by less than 10% of participants. While these relatively infrequently-occurring ACEs were included in all statistical analyses to highlight important trends and inform future research projects, the findings were interpreted with caution. The prevalence of all 13 ACE indicators is presented in Table 2.

In this cohort, the distribution of ACE-IQ was almost symmetric (median = mode = 5.0 and mean = 4.79). At time 1, but not at time 2, total ACEs were significantly associated with depressive symptoms (r = 0.23, *p* < 0.05) and perceived stress (r = 0.18, *p* < 0.05). Depressive symptoms and perceived stress were significantly correlated at both time points (time 1: r = 0.67, time 2: r = 0.33; both *p* < 0.05). Moreover, a significant correlation between assessments at times 1 and 2 was found for depressive symptoms (r = 0.19, *p* < 0.05) and perceived stress (r = 0.25, *p* < 0.05). Finally, depressive symptoms at enrolment were associated with lower pregnancy-related anxiety (r = −0.18, *p* < 0.05) and higher perceived stress (r = 0.21, *p* < 0.05) at follow-up. No other associations emerged as significant. All correlations are presented in Table 3.

Paired t-tests indicated that as depressive symptoms decreased, t (167) = −8.44, *p* < 0.001, perceived stress increased, t (167) = 4.60, *p* < 0.001, and pregnancy-related anxiety remained unchanged as pregnancy progressed. Of note, at the first assessment, 41 women (19.1%) were at high risk of depression (EPDS score >13) and 28 women (13.0 %) reported experiencing PRA (defined as three or more PRA items rated as “very true”). Among the 169 returning for follow up at 22–29 weeks’ GA, the percentages of women at high risk for depressive symptoms and with high PRA scores dropped to 1.4% (n = 3). Table 4 shows the mean and standard deviation of psychosocial distress scores at the two time points.

### 3.2. Hypothesis Testing

**Figure 1:**
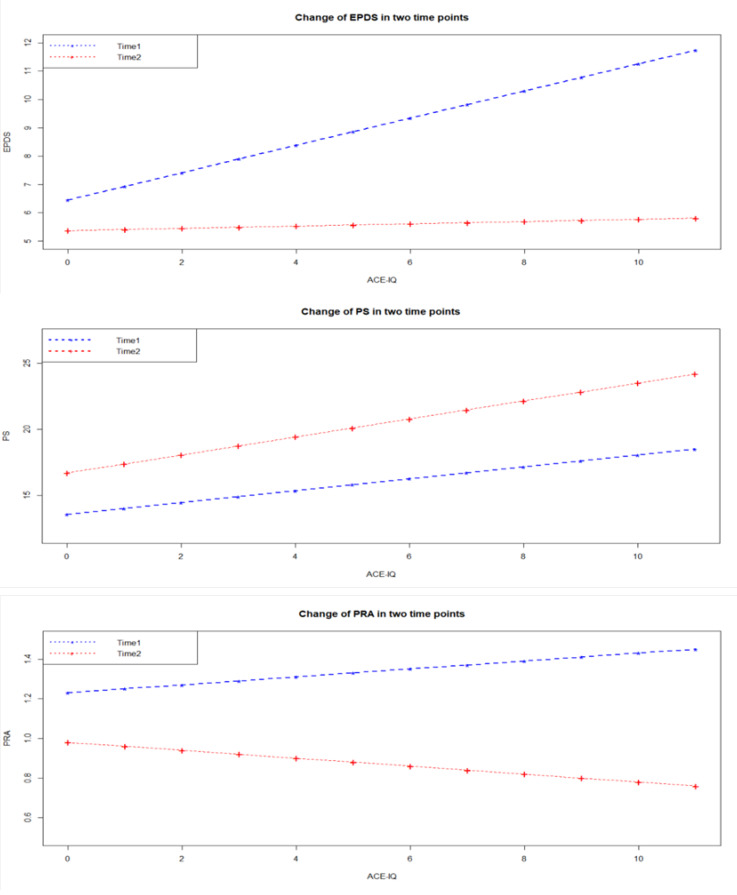
Change of Depressive Symptoms, Perceived Stress, and Pregnancy-Related Anxiety at two time points in pregnancy [5]. EPDS = Edinburgh Postnatal Depression Score, PS = Perceived Stress, PRA = Pregnancy-related anxiety.

The results from model 1, which compared the effect of the total ACE-IQ score on the three measures of distress (three models were computed: PRA, EPDS, PS), are reported in detail in Table 5 and graphically depicted in Figure 1.

The most significant result, perhaps, emerged for the EPDS model, which suggested significantly higher depressive symptoms with increased ACEs (*p* < 0.01; main effect ACE-IQ), and marginally lower depressive symptoms as pregnancy progressed (*p* = 0.05; main effect time). These main effects were subsumed by a significant time by ACE-IQ interaction (*p* < 0.01), suggesting that the positive association between the ACE-IQ and depressive symptoms was driven by associations early but not late in pregnancy. Similarly, significant ACE-IQ and time effects (both *p* < 0.01) were found in the model for perceived stress; however, the interactive term did not emerge as significant, indicating that perceived stress in pregnancy was higher with increased ACE-IQ irrespective of time, and that perceived stress was higher later compared to earlier in pregnancy, irrespective of ACE-IQ. No significant associations were observed in the model for pregnancy-related anxiety, although a marginal main effect of time, indicating lower anxiety later compared to earlier in pregnancy, deserves mention. Model 2 was fitted by adding covariates to model 1, but none of the individual main effects of the covariates or their interactions with the ACE-IQ score were significant in predicting EPDS, PS, or PRA, suggesting that sociodemographic factors did not influence our findings.

In the next step, models were rerun with individual ACE subscales (as opposed to the total ACE score) entered simultaneously, as predictors and non-significant factors drop using conditional likelihood ratio criterion. In the EPDS model, contact sexual abuse (*p* < 0.01) and parental death or divorce (*p* = 0.01) emerged as significant predictors, and a significant time effect also emerged (*p* < 0.01). However, the interaction effects between sexual abuse and time (*p* = 0.11) and between parental death/divorce and time (*p* = 0.13) on depression were not significant, suggesting that the association between contact sexual abuse and depressive symptoms, as well as the association between parental death/divorce and depressive symptoms, did not change during pregnancy.

In the perceived stress model, significant mentally ill household member effects (*p* = 0.01) and time effects (*p* < 0.01) were again found to be significant, but in contrast to model 1 using the total ACE-IQ scale, these main effects were subsumed by a significant interactive effect (*p* = 0.03), suggesting that the significant association between ‘presence of a mentally ill household member’ and perceived stress was driven by the findings at time 2. Finally, in the pregnancy-related anxiety model, the main effects for time and ‘presence of a mentally ill household member’ did not emerge as significant, but the interaction of the two terms did (*p* < 0.01). For each of the models for EPDS, PS, and PRA involving individual ACE-IQ indicators, we again explored if any of the covariates were moderators of the relationship in the model (model 4). We found again that none of the covariates served as moderating factors. The findings from these models are presented in Table 6.

## 4. Discussion

In this study, women with higher total ACE scores reported more depressive symptoms and more perceived stress compared to women with lower total ACE scores. No relationship between ACE and pregnancy-related anxiety was observed. Perhaps most interestingly, total ACEs in this study were associated with depressive symptoms early but not late in pregnancy, an effect that was not significant but trended in that direction for two ACEs—sexual abuse and parental death or divorce. Pregnancy is a time of significant physiological and psychosocial change, and it is possible that windows of vulnerability exist in which previous trauma is more likely to contribute to depressive symptoms compared to other time points. Previous research suggests that there is a window of vulnerability around 25 weeks’ GA in which pregnancy-related physiological changes in maternal stress hormones are predictive of maternal post-partum depressive symptoms [40,41], and that these changes can be modulated by social support [40]. Our current findings add to these observations by suggesting that this might also be a time during which the effects of previous trauma on pregnant women’s mental health may also become more pronounced.

The finding of an overall association between ACEs and increased maternal depressive symptoms and perceived stress adds to a growing number of studies that report on the adverse effects of ACEs on maternal mental health. Associations between ACEs and perceived stress in pregnancy have also been previously reported in High Income Countries (HIC) [42], and one study found paternal ACEs associated with fathers’ depressive symptoms and pregnancy-related anxiety [43].

When individual ACEs were considered, based on the trend towards significance, sexual abuse and parental death/divorce might play a more important role in the context of pregnancy than other ACEs in this setting. The experience of early parental loss or divorce might become more salient once women prepare to become parents themselves. Regarding childhood sexual abuse, associations with depressive symptoms in pregnancy have been previously reported [44], and pregnancy itself is associated with triggering experiences that can be reminders of previous abuse [45].

This study was conducted at a private not-for-profit health institution which in itself limits the generalizability of the findings to diverse populations, and in keeping with this context, we observe that the majority of women in our sample were well educated and had a relatively high household family income which is not the case for the majority of the region. Use of self-report questionnaires that were developed in high-income countries and utilized in a low to middle income country presents a limitation. It is unclear whether the questions asked are equally applicable and interpreted comparably. We recommend that future studies should further validate questionnaires measuring psychosocial distress among pregnant women in a local context.

## 5. Conclusions

Higher total ACEs were positively associated with depressive symptoms and perceived stress during pregnancy. Contact sexual abuse and parental death or divorce in particular were associated with depressive symptoms in this context. Future studies are needed to take into account other mental disorders in addition to anxiety, stress, and depressive disorders that may have an impact on pregnancy outcomes and also further validate the utility of the EPDS tool in local settings.

## Figures and Tables

**Table 1:** Participant characteristics at initial study time-point (12–19 weeks’ Gestational Age) (n = 215).

Characteristic	Value
**Age M (SD)**	**30.55 (4.32)**
Range	22–47
**Education n (%)**	
Secondary/High school completed	9 (4.2)
College/University completed	160 (74.4)
Post graduate degree	46 (21.4)
**Marital Status n (%)**	
Married	174 (80.9)
Not married	41 (19.1)
**Working status n (%)**	
Government employee	34 (15.8)
Non-government employee	105 (48.8)
Self-employed	50 (23.3)
Student	10 (4.7)
Homemaker	9 (4.2)
Unemployed (able to work)	7 (3.3)
**Annual Family Income n (%)**	
KES 40,001–100,000	36 (16.7)
KES 100,001–200,000	86 (40.0)
More than KES 200,001	87 (40.5)
**Ethnicity n (%)**	
Kikuyu	93 (43.3)
Kalenjin	10 (4.7)
Kamba	20 (9.3)
Luhya	23 (10.7)
Luo	20 (9.3)
Other	49 (22.8)
**Health Behaviors n (%)**	
**Alcohol**	
Never	209 (97.2)
Monthly or less	5 (2.3)
2–3 times per week	1 (0.5)
**Smoking**	1 (0.5)
**Pica**	3 (1.4)

**Table 2:** Prevalence of Adverse Childhood Experiences (n = 215).

Childhood Experience	n (Prevalence %)
**Neglect**	
Emotional neglect	181 (84.2)
Physical neglect	12 (5.6)
**Psychological Distress of Family Members**	
Alcohol or drug abuse in household	24 (11.2)
Incarcerated household member	7 (3.3)
Mentally ill household member	5 (2.3)
Parental death or divorce	54 (25.1)
**Violence at Home**	
Household member treated violently	140 (65.1)
Emotional abuse	109 (50.7)
Physical abuse	167 (77.7)
**Sexual Violence**	
Contact sexual abuse	38 (17.7)
**Violence in Community**	
Bullying	94 (43.7)
Community violence	171 (79.5)
Collective violence	28 (13)

**Table 3:** Pearson Correlations between psychosocial distress scores at enrolment and follow-up.

		Scores at Enrolment				Scores at Follow-Up		
Time		ACE-IQ Score	Anxiety	Depressive Symptoms	Stress	Anxiety	Depressive Symptoms	Stress
Enrolment	ACE-IQ binary	1.00						
	Anxiety	0.02	1.00					
	Depressive Symptoms	0.23 *	0.05	1.00				
	Perceived Stress	0.18 *	0.02	0.67 *	1.00			
Follow Up	Anxiety	−0.04	0.07	−0.18 *	−0.07	1.00		
	Depressive Symptoms	0.01	−0.07	0.19 *	0.14	0.03	1.00	
	Perceived Stress	0.07	−0.08	0.21 *	0.25 *	−0.05	0.33 *	1.00

* Significant at *p* = 0.05 or lower.

**Table 4:** Descriptive Statistics for Psychosocial Distress Measures at the Two Time Points.

	Time 1 (n = 215)	Time 1 * (n = 169)	Time 2 (n = 169)	Paired Comparison (T2-T1) (n = 169)		
Scale	Mean (SD)	Mean (SD)	Mean (SD)	Mean (Std Err)	t (df = 167)	*p*-Value
Anxiety	1.3 (1.33)	1.3 (1.36)	1.1 (1.04)	−0.21 (0.13)	−1.70	0.091
Depression	8.6 (4.33)	8.8 (4.37)	5.6 (2.78)	−3.04 (0.36)	−8.44	<0.001
Stress	15.7 (5.21)	15.8 (5.21)	17.8 (4.27)	2.07 (0.45)	4.60	<0.001

* Excludes women who did not return for the follow up visit. Time 1 = 12–19 weeks’ GA, Time 2 = 22–29 weeks’ GA = Gestational Age, T1 = Time 1, T2 = Time 2.

**Table 5:** Effect of Total ACE-IQ Score on Psychosocial Distress in Pregnancy.

Model Predictors	Anxiety (PRA)		Depression (EPDS)		Perceived Stress (PS)	
	Estimate	*p*-Value	Estimate	*p*-Value	Estimate	*p*-Value
Constant	1.23 (0.23)	<0.01	6.46 (0.73)	<0.01	13.57 (0.88)	<0.01
Time	−0.25 (0.3)	0.05	−1.09 (0.83)	0.05	3.14 (1.07)	<0.01
ACE-IQ	0.02 (0.04)	0.72	0.48 (0.14)	<0.01	0.45 (0.17)	0.01
Time * ACE-IQ	−0.04 (0.06)	0.48	−0.44 (0.16)	<0.01	0.23 (0.21)	0.12

* Time = Time period (0 at 12–19 weeks’ GA and 1 at 22–29 weeks’ GA = Gestational Age).

**Table 6:** Effect of Individual ACE-IQ Score Indicators on Psychosocial Distress in Pregnancy.

	Estimate	*p*-Value
**(a) PRA**		
(Intercept)	1.29 (0.09)	<0.01
Time	−0.20 (0.12)	0.10
Mentally ill household member	0.91 (0.61)	0.14
Time * Mentally ill household member	−2.00 (0.74)	0.01
**(b) EPDS**		
(Intercept)	7.96 (0.36)	<0.01
Time	−2.58 (0.41)	<0.01
Contact sexual abuse	2.24 (0.76)	<0.01
Parental death or divorce	1.67 (0.67)	0.01
Time * Contact sexual abuse	−1.41 (0.88)	0.11
Time * Parental death or divorce	−1.16 (0.76)	0.13
**(c) PS**		
(Intercept)	15.61 (0.35)	<0.01
Time	2.19 (0.43)	<0.01
Mentally ill household member	6.19 (2.33)	0.01
Time * Mentally ill household member	5.59 (2.62)	0.03

EPDS = Edinburgh Postnatal Depression Score, PS = Perceived Stress, PRA = Pregnancy-related anxiety, * Time = Time period (0 at 12–19 weeks’ GA and 1 at 22–29 weeks Gestational Age).
